# Perinatal outcomes of pregnancies with borderline versus normal amniotic fluid index

**Published:** 2013-09

**Authors:** Maryam Asgharnia, Roya Faraji, Fatemeh Salamat, Babak Ashrafkhani, Seyedeh Fatemeh Dalil Heirati, Samira Naimian

**Affiliations:** 1 *Reproductive Health Research Center, Department of Obstetrics and Gynecology, Guilan University of Medical Sciences, Rasht, Iran. *; 2 *Vice-chancellorship for Research, Guilan University of Medical Sciences, Rasht, Iran.*; 3 *Guilan University of Medical Sciences, Rasht, Iran.*

**Keywords:** Borderline, Amniotic fluid index, Pregnancy outcome, Pregnancy complication.

## Abstract

**Background:** Amniotic fluid is an indicator of placental function on the fetal development. The amniotic fluid index is the most commonly used method of measuring amniotic fluid.

**Objective: **The purpose of this study was to compare the pregnancy outcomes of a borderline versus normal AFI.

**Materials and Methods:** This cross-sectional study was carried out on a total of 235 pregnant women referred to Alzahra Medical Center between 2009-2011. Women with a singleton pregnancy in third trimester were enrolled into this study; of these subjects, 141 cases were in normal AFI group and 94 cases in borderline AFI group. Adequate information was obtained from the patients' medical record and the groups were compared on maternal and fetal complications. Data analysis was performed by using SPSS.

**Results: **The mean maternal age in borderline AFI group was 25.96±5.92 years and in normal AFI group was 27.88±6.5 years (p=0.023). Maternal outcomes such as preterm delivery and labor induction in women with borderline AFI were considerably higher than those in normal group (p=0.01 and p=0.001). There were no significant differences between the two groups in terms of high blood pressure, preeclampsia, diabetes and neonatal respiratory distress. The borderline AFI group had higher rate of neonatal complications such as Apgar score of less than 7 (p=0.004), IUGR (0.0001), LBW (0.001), and crucial need to NICU (0.003).

**Conclusion:** Findings indicated that there are statistical differences between adverse outcomes in borderline AFI group and normal group.

This article extracted from M.D. thesis. (Samira Naimian)

## Introduction

Antenatal test is done to evaluate fetus health and the risk of adverse outcomes during the course of a pregnancy (1). Amniotic fluid is an important part of pregnancy which plays a vital role in the normal growth of the fetus and, promotes muscular-skeletal development and allows for easier fetal movement.

Amniotic fluid assessment is an essential part of evaluation of fetus health in terms of fetal distress, meconium aspiration, caesarean and fetal mortality (2). The assessment of amniotic fluid volume is very crucial for the survival of the fetus and the Amniotic Fluid Index (AFI) is the most common way for the estimation of amniotic fluid volume which is performed by ultrasound method (3, 4). Studies have revealed that AFI is an accurate criterion for estimating adequate placental function (5). Amniotic fluid volume varies with gestational age, rising to a plateau between 22-39 weeks of gestation and reaching 700 and 800 ml, which correspond to an AFI of 14-15 cm (6, 7). Any decrease or increase in the volume of amniotic fluid leads to pregnancy complications (2).

In most studies oligohydramnios has been defined as an AFI of 5 cm or less and its associated maternal and fetal complications are proven (8, 9). However, there are different views about the range of borderline AFI. In a study done by Phelan *et al* borderline AFI is defined between 5 and 8 cm (8, 10, 11). Also, Gumus and Miller have defined a borderline AFI as an AFI of 5.1-10 (12, 13). 

In spite of different views on borderline AFI in different studies, there are, also, different views about its function and influence on maternal and fetal complications and medical care for fetus health. In most reported studies, the pregnancies with borderline AFI of 5-10 cm have shown outcomes such as non-reactive non-stress tests, fetal heart rate (FHR) deceleration, meconium aspiration, immediate caesarean delivery, low Apgar score, LBW, NICU admission and SGA in comparison with control subjects with normal amniotic fluid level (8.1-18 cm) (8-10, 14-16). Also the low amniotic index may increase the operative delivery rate (3). 

Also, according to Luo *et al* the pregnancy outcomes of a borderline versus normal AFI suggested no difference in the incidence of fetal distress or neonatal mortality, but the rate of caesarean delivery in borderline AFI was reported higher than the rate in normal cases. They evaluated 196 trails of labor with a borderline AFI (5.^1^-^8^) and 200 women with normal AFI (8.^1^-^18^) (17). Meanwhile, in another study, oligohydramniosis was shown to be associated with pregnancy complications but the diminished amniotic fluid volume doesn't seem to have any noticeable effect on anticipating the outcomes (18). 

Therefore, despite so many studies, the predicative accuracy of borderline AFI for an adverse pregnancy outcome is not absolutely definite and prenatal assessment in women with borderline AFI is not recommended (1). But most findings suggest that even though there is insufficient evidence or indication to begin antenatal testing, the results of borderline AFI should be carefully interpreted, and a diagnostic sonography should be used to confirm SGA and IUGR (1). More study is needed because of contradictions and insufficient evidence about delivery based on a borderline AFI. The current study aims to compare pregnancy outcomes of a borderline versus normal AFI after controlling confounding variables.

## Materials and methods

This cross-sectional study was conducted on pregnant women referred to Alzahra Medical Center between 2009 and 2011. Women with a singleton pregnancy who were in third trimester (≥28 weeks) were included in this study and outcomes were studied retrospectively after delivery. 

The gestational age was calculated from the first day of the last menstrual period or calculated by sonography before 12 weeks of gestation. Exclusion criteria were premature rupture of membranes, meconium aspiration, uterine anomalies and vaginal bleeding. Sample size was estimated with regard to the occurrence of intrauterine growth restriction in patients with borderline AFI in previous study (12) (power 80%, p=0.05). in total 94 subjects with borderline AFI in case group and 141 subjects with normal AFI in control group were considered.

Sonographic report made by one physician was used in order to determine the accuracy of borderline AFI. Women after childbirth were selected for the study from the Department of Obstetrics and Perinatal Intensive Care Unit for control group and case group, respectively. Normal amniotic fluid volume and borderline amniotic fluid were defined as 10<AFI<24 and 5<AFI<10, respectively, and at least two sonographic assessments after 28 week were required to confirm borderline AFI (12). 

Adequate information was obtained by the data within the patients' medical record and factors such as gestational age, number of births, number of pregnancies, pregnancy with diabetes, high blood pressure(blood pressure >140 mmHg systolic and/or >90 mmHg diastolic, twice, at least 6 hours apart or not more than one week apart), preeclampsia, pregnancy and prenatal outcomes (Intrapartum fetal distress, preterm birth or birth under 37 weeks, induction, 5-minute Apgar score, birth weight, ICU admission and fetal growth restriction) were analyzed and recorded. The approval letter was obtained from Guilan University of Medical Sciences Ethic Committee.

**Statistical analysis**

Data analysis was performed by using SPSS version16, and Independent T-test was used to make a comparison between quantitative variables of two different groups, and Chi-square or Fisher-Exact-test for categorical variables. We used Logistic regression model to adjust for confounding variables in addition to AFI for each adverse outcome. 

## Results

A total of 235 eligible women were enrolled into this study. Of these subjects, 94 were in borderline AFI group and 141 were in normal AFI group. Baseline characteristics of the study participants are illustrated in table I. There were no statistically significant differences between the two groups for gravity, parity, gestational diabetes mellitus, hypertension and preeclampsia (Table I). In control group, the mean of mother age (Y) was significantly higher than in the border AFI group (р=0.02, Table I). 

Occurrence of preterm labor and Induction of labor as maternal outcome in border AFI group were significantly higher than in the control group (р<0.001, p=0.017 respectively, Table II). Among neonatal outcomes, apgar score<7 at 5 min, neonatal intensive care unit (NICU) admission, intrauterine growth restriction (IUGR) and low birth weight (LBW) in Border AFI group were significantly higher than in the control group (p<0.01, Table III). 

A total of 13.8% of border AFI group and 6.4% of control group showed respiratory distress syndrome (RDS); But this was not statistically significant (p=0.067, Table III). To adjust for confounding variables, we include mother age in addition to AFI in the logistic regression model as independent factor. The risk of each adverse outcome showed an obvious increase in association with borderline AFI after adjusting for maternal age (Table IV).

**Table I:** Background characteristics of study groups

			**Borderline AFI (n= 94)**	**Normal AFI (n= 141)**	**p-value**
Mother age			25.96±5.92	27.88±6.5	0.02
Gravity					
		1	58 (61.7)	72 (51.1)	0.14
		2 and more	36 (38.3)	69 (48.9)	
Parity					
	0		64 (68.08)	82 (58.15)	
	1		23 (34.04)	46 (32.62)	0.23
	2 and more		7 (7.44)	13 (9.2)	
Gestational DM			4 (4.3)	9 (6.38)	0.57
HTN			8 (8.51)	8 (5.67)	0.43
Preeclampsia			6 (6.4)	5 (3.5)	0.35

Data are mean ± SD or n (%) as appropriate. AFI indicates amniotic fluid index; DM: diabetes mellitus; HTN: hypertension.

p<0.05 is considered significant.

Independent t-test was used for analysis of quantitative variables and chi-square test for categorical variables.

**Table II:** Comparison of maternal outcomes in study groups

	**Borderline AFI [n= 94 (%)]**	**Normal AFI [n= 141(%)]**	**p-value**
Preterm labor (<37week)	38 (40.4)	21 (14.9)	0.0001
Induction of labor	21 (22.34)	15 (10.6)	0.017

Data are frequency (percent).

P<0.05 is considered significant.

Chi-square test was for analysis of categorical variables.

**Table III:** Comparison of neonatal outcomes in study groups

	**Borderline AFI [n= 94 (%)]**	**Normal AFI [n= 141 (%)]**	**p-value**
RDS	13 (13.8)	9 (6.4)	0.067
AS <7 at 5 min	19 (20.2)	10 (7.1)	0.004
NICU admission	14 (14.9)	5 (3.5)	0.003
IUGR	25 (26.6)	5 (3.5)	0.0001
LBW	45 (47.87)	29 (20.56)	0.0001

Data are frequency (percent). RDS indicates respiratory distress syndrome; AS: apgar score; NICU: neonatal intensive care unit; IUGR: intrauterine growth restriction; LBW: low birth weight.

p<0.05 is considered significant.

Chi- square test was used for analysis of categorical variables.

**Table IV:** The adjusted OR for each prenatal outcome for AFI

							**Adjusted OR**	**CI (95%)**	**p-value**
IUGR									
		AFI							
		Normal					1		
		Borderline					9.85	(3.61-26.86)	0.001
NICU admission									
	AFI								
	Normal						1		
	Borderline						4.76	(1.65-13.7)	0.004
Preterm labor									
			AFI						
			Normal				1		
			Borderline				3.87	(2.08-7.20)	0.001
AS<7 at 5 min									
				AFI					
				Normal			1		
				Borderline			3.32	(1.46-7.51)	0.004
LBW									
					AFI				
					Normal		1		
					Borderline		3.25	(1.83-5.76)	
Induction									
						AFI			
						Normal	1		
						Borderline	2.56	(1.25-5.26)	0.01

OR indicates odds ratio; CI, Confidence Interval; IUGR, intrauterine growth restriction ; NICU, neonatal intensive care unit; AS: apgar score; LBW: low birth weight.

p<0.05 is considered significant.

Logistic regression (forward stepwise (wald) ) was used to adjust mother age.

## Discussion

Many studies have been done to show the association of a borderline amniotic fluid index with some adverse perinatal outcomes and, in most findings, the occurrence of maternal and fetal complications was reported more often in pregnancies with borderline AFI than in those with normal AFI (1, 16).

However, there were no specific perinatal cares or other care protocols for these patients and that could be because of different reasons such as the variations in the study designs, the likelihood of a borderline index varied from 6-44% and 25-35% and the absence of receiver-operating characteristic curve to determine the thresholds of adverse outcomes, and therefore, more research will be required to find out the effect of AFI on adverse pregnancy outcome (19-21).

So in the present study, the maternal and fetal complications in women with borderline AFI were compared with complications in those with normal AFI among 235 pregnant women in Alzahra Hospital which confirmed the increased adverse perinatal outcomes in women with borderline AFI. Findings indicated that maternal outcomes such as preterm delivery and labor induction in women with borderline AFI were considerably higher than those in normal group and that was consistent with the findings in some other studies with the same results (3, 12, 16, 22).

In addition, the borderline AFI group had higher rate of neonatal complications such as Apgar score of less than 7, IUGR, LBW, and crucial need to NICU and there were similarities between the findings of this research and the existing work of others. For example, Petrozella *et al* reported the rate of caesarean 24% and the birth weight below the third percentile 21%; or Banks considered the likelihood of IUGR up to 4 times greater, and Gumus *et al* found a higher rate of IUGR, LBW, Apgar score of less than 7 at 5 minutes, and NICU admission among women with borderline AFI which were in accordance to our results (3, 4, 13, 16).

In current research, no significant difference was found in the incidence of respiratory distress between the two groups, whereas there was a significant difference among the patients with gestational age between 28 and 32 weeks which could be because of premature births in this group. Also, in some other studies, there were increased incidences of respiratory distress among infants in the borderline AFI group and it was probably because borderline AFI was evaluated at lower gestational age, and as a result, prematurity was associated with respiratory distress (3, 12).

The present study showed no statistical differences between the ratios of gravidity and parity in the two study groups; whereas, in Gumus *et al* and Voxman *et al* study, the groups were similar with respect to maternal age, gravidity and parity (4, 12). Also, the present study analysis showed no significant differences between the two groups in terms of high blood pressure, pre-eclampsia and diabetes for the mother and that was consistent with the results of Gumus *et al*. However, **t**here were a significantly higher percentage of NICU admission in patients with normal AFI than in those with oligohydramnios. That appeared to be attributable to the higher percentage of women with diabetes in the normal AFI group. Then reanalysis of their data with exclusion of the diabetic patients resulted in no significant difference between the two groups (4). 

In our institution, infants with apgar less than 7 at 1 and 5 minute are routinely observed in the NICU after delivery and this may contribute to the higher rate of admission in NICU. Therefore, because of the fact that the findings in this study reinforces the increased pregnancy complications in women with borderline AFI, and because of the lack of a definite care protocol to care the patients, the physicians recommend that the patients have twice weekly sonography assessment to evaluate AFI and to permanently monitor the patients for IUGR and SGA and to take all necessary measures in order to avoid adverse perinatal complications (1, 23). Further studies are warranted to confirm the effect of AFI on pregnancy outcome.

## Conclusion

In conclusion, due to such adverse outcomes mentioned in patients with borderline AFI and because there is no sufficient evidence and specific decision about delivery based on a borderline AFI, there should be a close observation and antepartum surveillance for them. Also further studies with prospective design are needed.

## Conflict of interest

The authors report no conflict of interest related to this study.
